# Supplementary material for: Bile canaliculi formation in primary hepatocytes requires α1β1 integrin-dependent adherens junction re-organization
Source: J Cell Sci. 2025 Dec 5;138(23):jcs264412. doi: 10.1242/jcs.264412 (PMC12752502; doi:10.1242/jcs.264412)
Supplement: Supplementary information [file joces-138-264412-s1.pdf]

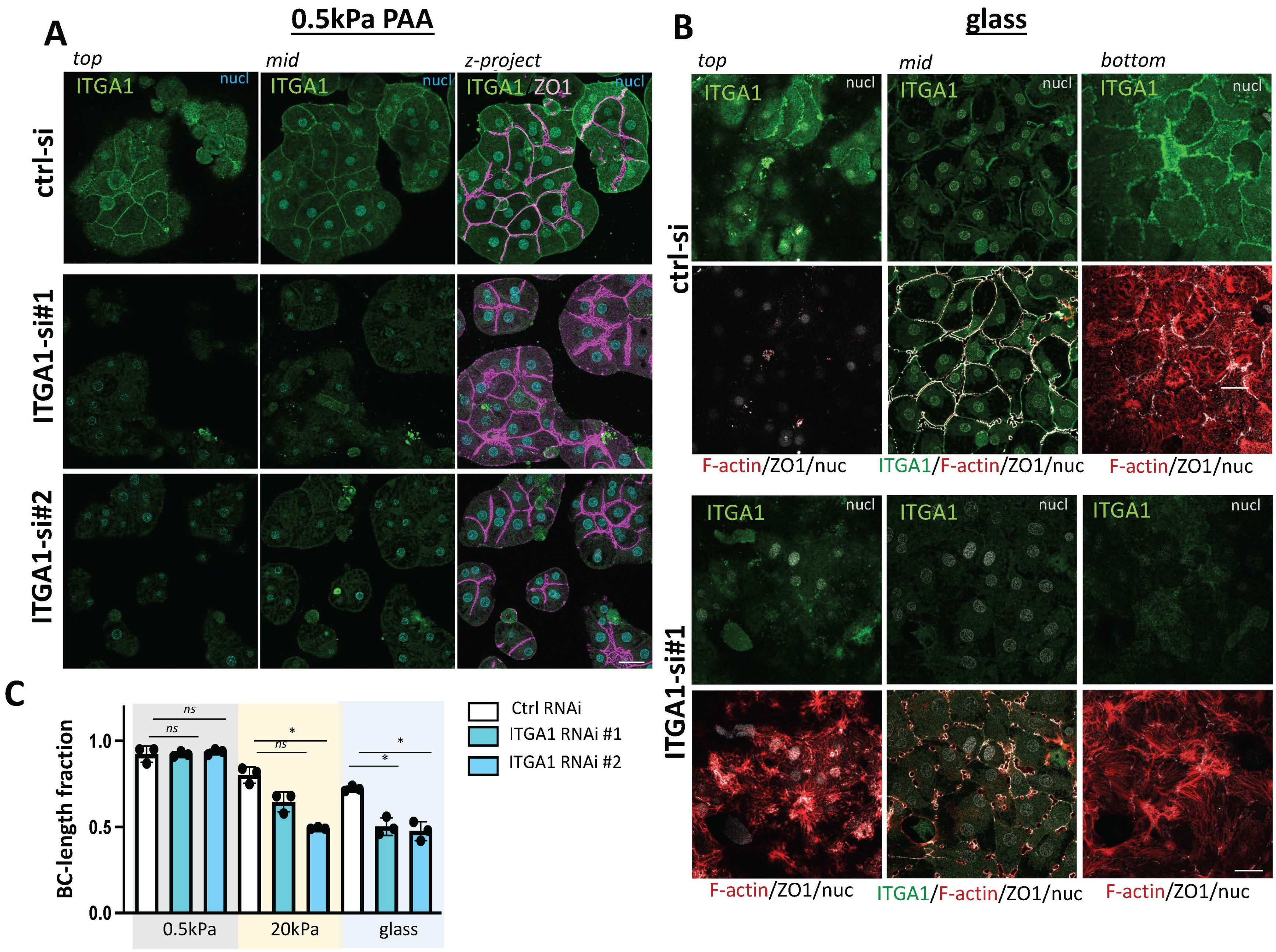

Fig. S1. siRNA-mediated α1/ITGA1 depletion reduces α1β1 -labeling in cultures on soft and rigid matrix, but affects BC-length only on rigid matrix

Upon plating on either coll-hydrogel of 0.5kPa (**A,C**), 20kPa(**C**) or coll-coated glass (**B,C**) hepatocytes were transfected with one of two ITGA1-siRNAs (si#1 or #si2) or ctrl siRNA, overlaid with collagen 20h later and cultured for 3d. Cells were fixed and labeled for the indicated markers. Note that ITGA1-si samples feature comparable loss of lateral α1β1 -labeling on glass and 0.5kPa-PAA (A,B), but a disrupted BC-network only on rigid matrix (B), where F-actin fibers at the overlay domain were apparent upon ITGA1si but not in controls. **C)** BC-length fractions in ctrl and ITGA1si for the different culture substrates are presented as means w SD; * $p<0.05$, One Way ANOVA with multiple comparisons. scale bar = 50µm.

Journal of Cell Science • Supplementary information

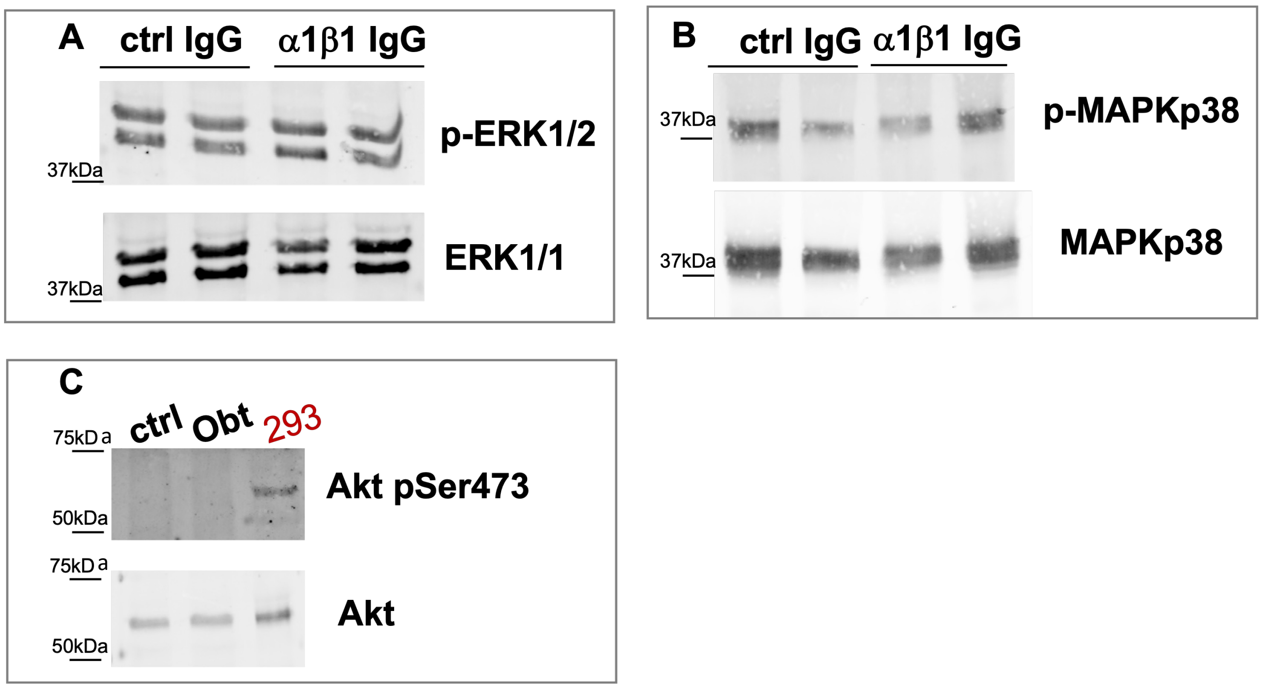

Fig. S2. α1β1-inhibition does not affect ERK, p38 and AKT activities

Representative images of ERK/pThr102/Tyr104-ERK (**A**) MAPKp38/pThr180/Tyr182-MAPKp38 (**B**) and Akt/pS473-AKT (**C**) levels in ctrl IgG or α1β1 IgG treated 3d-overlay cultures on glass. Note the absence of p-AKT in hepatocytes; HEK293 cells were used as positive control for the antibody. The means with SD of the intensity ratio between ctrl / α1β1 IgG for phospho-ERK1/2 was 0.93 $\pm$ 0.13 (n=6), and for phospho-p38 0.92 $\pm$ 0.39 (n=5); two tailed paired t-tests between ctrl and α1β1 IgG intensities yielded p=0.24 and p=0.43, respectively.

Journal of Cell Science • Supplementary information

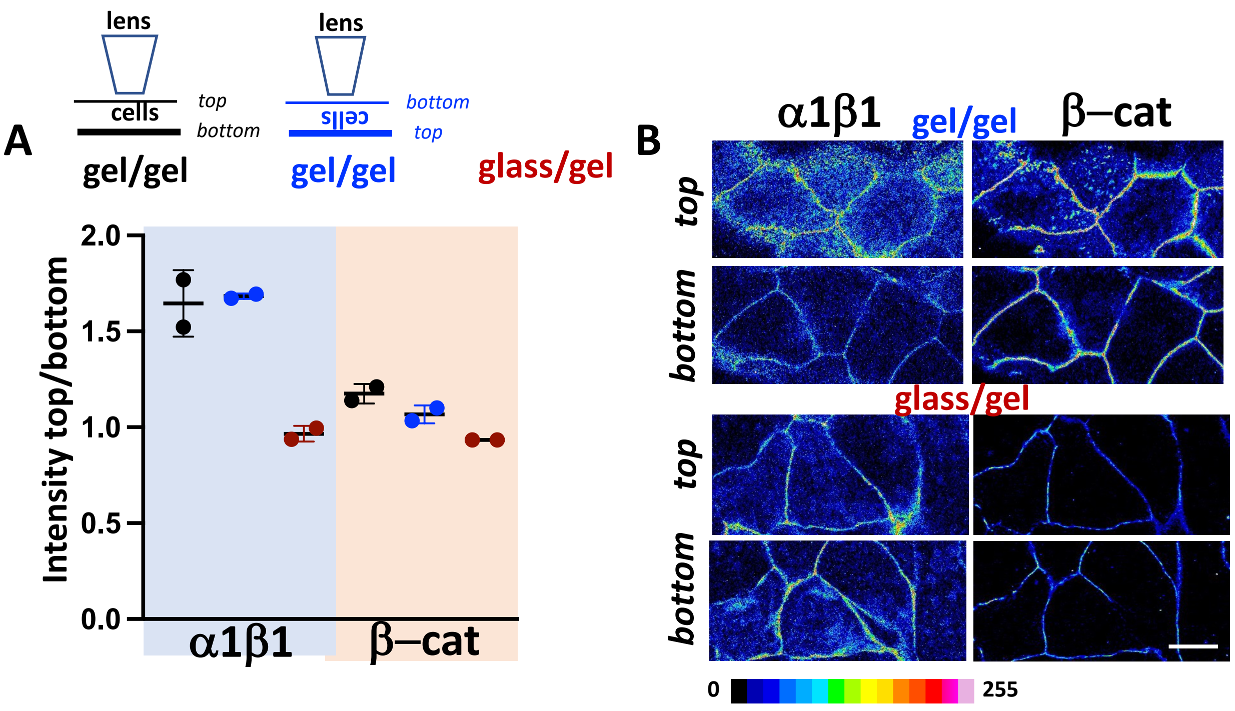

Fig. S3. In polarized coll gel sandwich cultures α1β1 is enriched near the overlay domain

A) Ratios of α1β1 and β-catenin mean lateral membrane fluorescence intensities measured near the overlay domain to that measured near the plating domain and **B**) examples of corresponding LUT images. Cells were plated on either collagen gel (gel/gel) or on coll-I-coated glass (glass/gel) and cultured for 3d upon collagen overlay. Black and blue data points correspond to upside-up (black) and upside-down (blue) mounting of coverslips, such that the microscope lens was either close to the top/overlay domain (black) or to the bottom/plating surface (blue) as illustrated schematically in A. Note that the two modii of coverslip mounting yielded similar ratios, indicating that the cells were within the focal distance of the lens. Note also, that no labeling asymmetry was observed for b-catenin on coll-I gels or for a1b1 on glass, suggesting that a1b1 enrichment at the top domain is not a technical artifact. Scale bar = 50µm.

Journal of Cell Science • Supplementary information

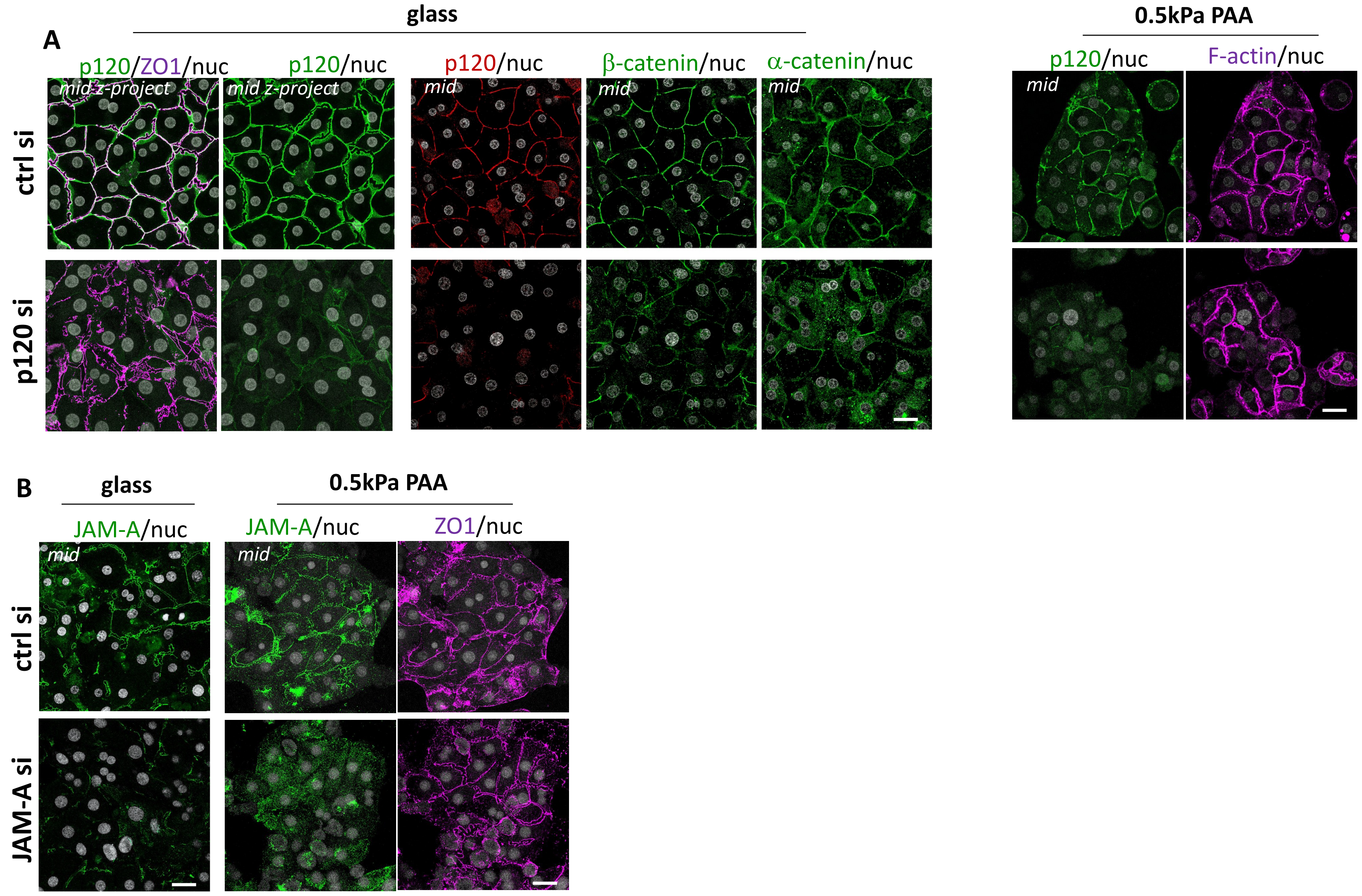

Fig. S4. cell-cell junction markers upon p120 and JAM-A depletion

Upon plating on coll-coated glass or on 0.5kPa PAA hydrogels hepatocytes were transfected with either p120 (**A**) or JAM-A (**B**) siRNA or with ctrl siRNA, overlaid with collagen 16h later and cultured for 3d. Cells were fixed and labeled for the indicated markers. Depletion was comparable on both substrates. Note in **A**) that in addition to loss of p120 from lateral membranes, membrane staining of α-catenin and β-catenin also decreased. ZO1 and nuclear labeling of the images in the p120/ZO1/nuc panels in A (glass) are also shown in Fig. 5A.

Journal of Cell Science • Supplementary information

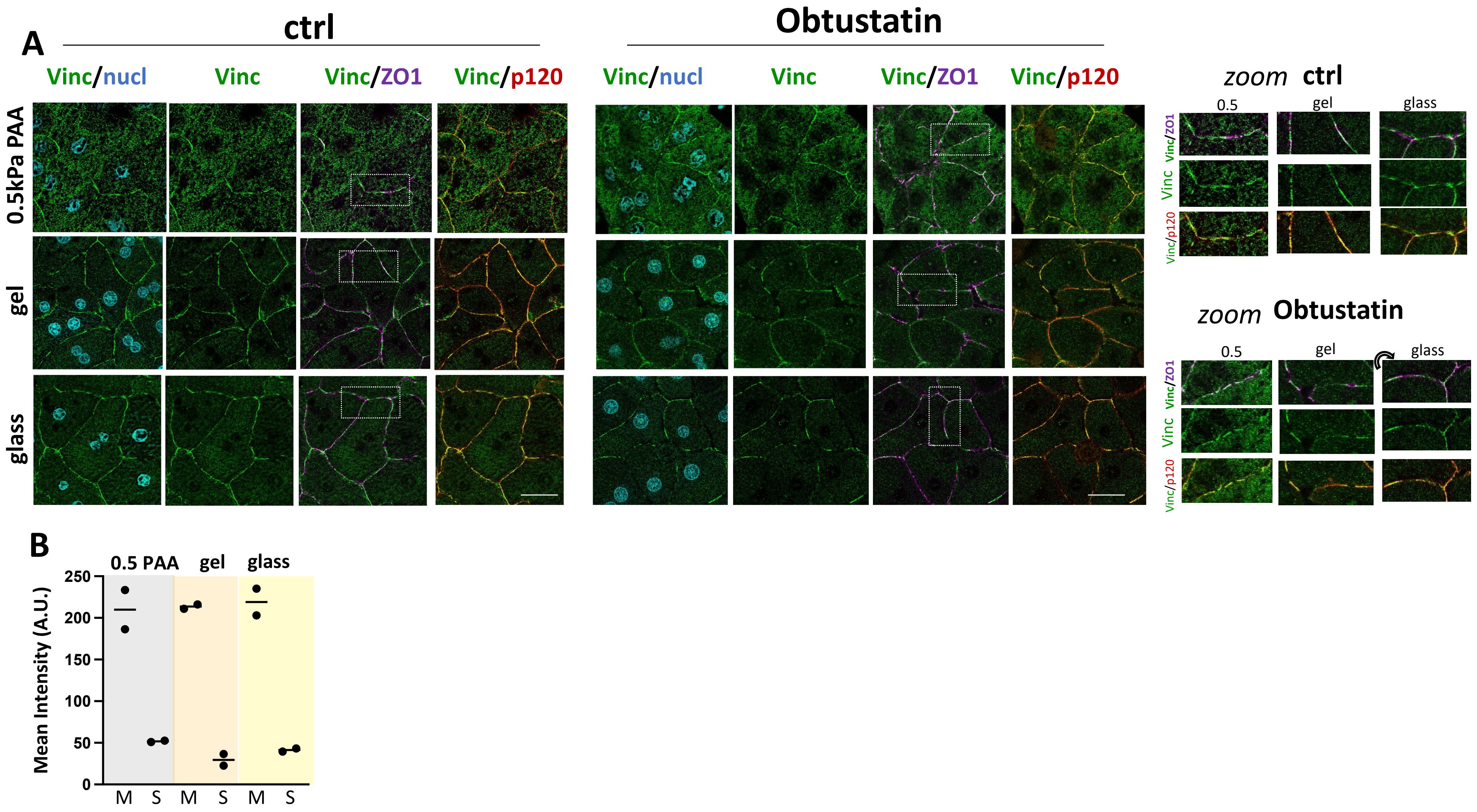

Fig. S5. Vinculin localizes to the ZA regardless of matrix rigidity or α1β1 inhibition

A) control and Obtustatin-treated cultures were plated on either coll-coated glass, collagen gel or coll-functionalized 0.5kPa PAA and overlaid with coll-gel for 3d; shown are confocal mid-sections where Vinculin colocalizes with p120 catenin next to ZO1; boxed fields are shown as zoomed inserts at right. Note that Vinculin is present at the lateral domain regardless of Obtustatin treatment and of plating substrate; **B)** Mean fluorescence intensities of membrane-associated Vinculin (M) at the ZA compared to soluble (S) Vinculin in the cytoplasm obtained in two experiments from control cultures on the indicated matrices; Note that ZA-associated Vinculin levels (M) in cells plated on 0.5kPa PAA, gel and glass are comparable. See *Methods* for analysis details. Scale bars = 50µm.

Journal of Cell Science • Supplementary information

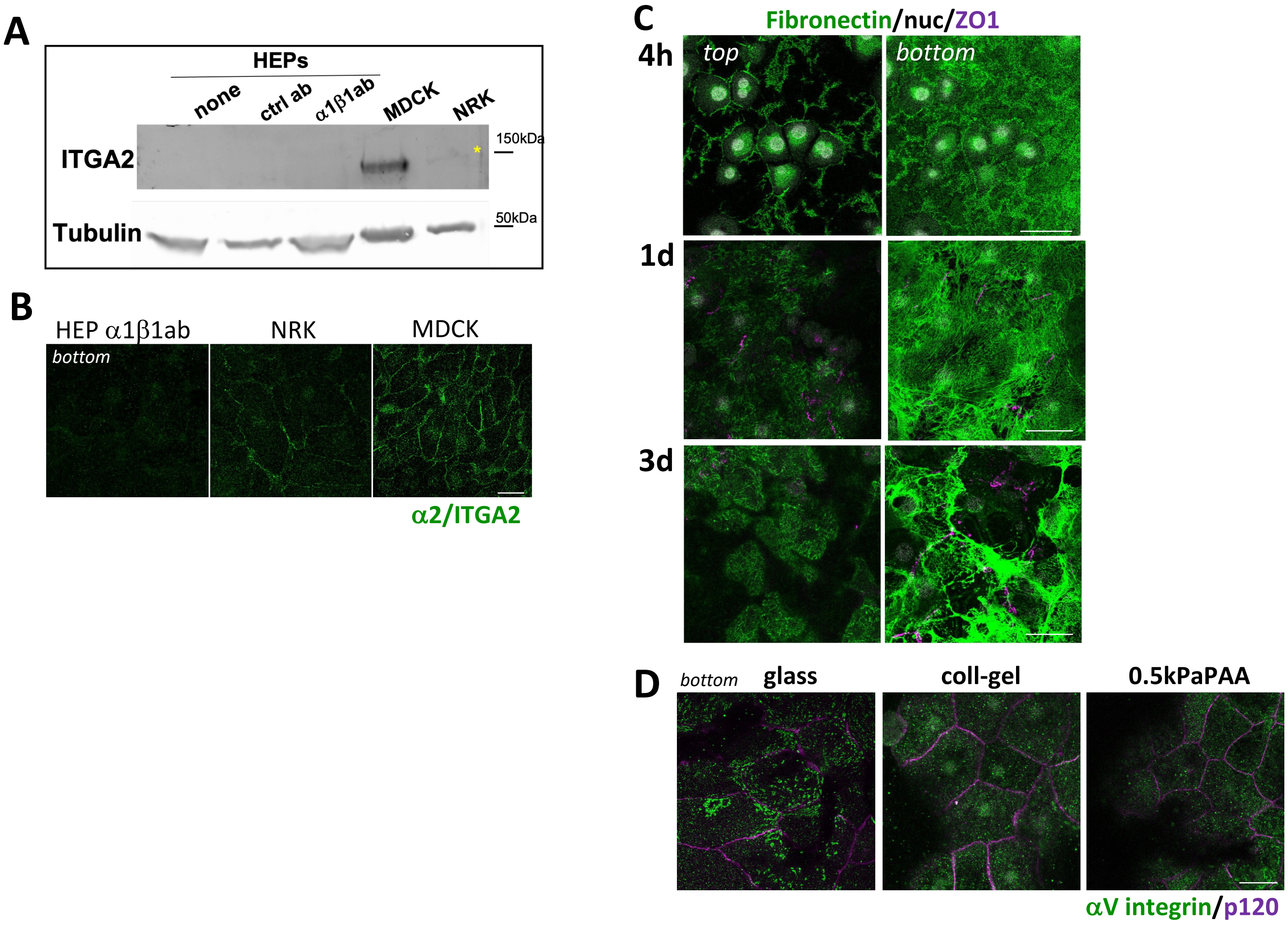

Fig. S6. Integrin and fibronectin expression

A,B) hepatocytes do not express α2β1: α2/ITGA2 immunoblot (A) and IF (B) using a polyclonal antibody in rat hepatocyte collagen sandwich cultures treated with ctrl or α1β1 -ab; canine MDCK and rat NRK cells were analyzed for comparison; asterisk in A) points to weak labeling in NRK cells, which is also apparent by IF; **C)** fibronectin labeling at top and bottom planes of hepatocytes 4h upon plating onto coll-coated glass, and 1d and 3d upon collagen overlay; **D)** cultures in coll-gels do not form aV-positive adhesions; scale bars = 50µm

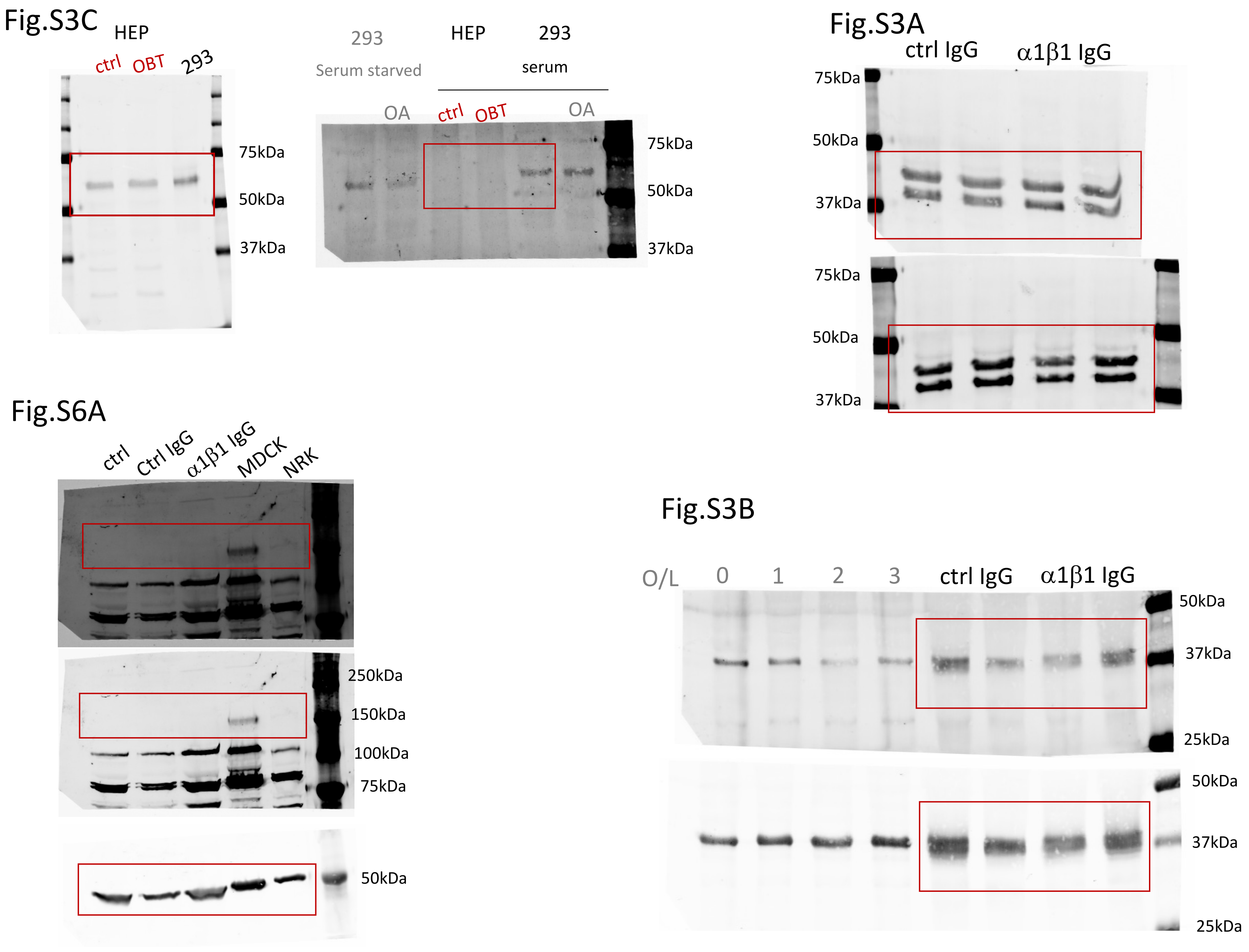

Fig. ST. Blot transparency

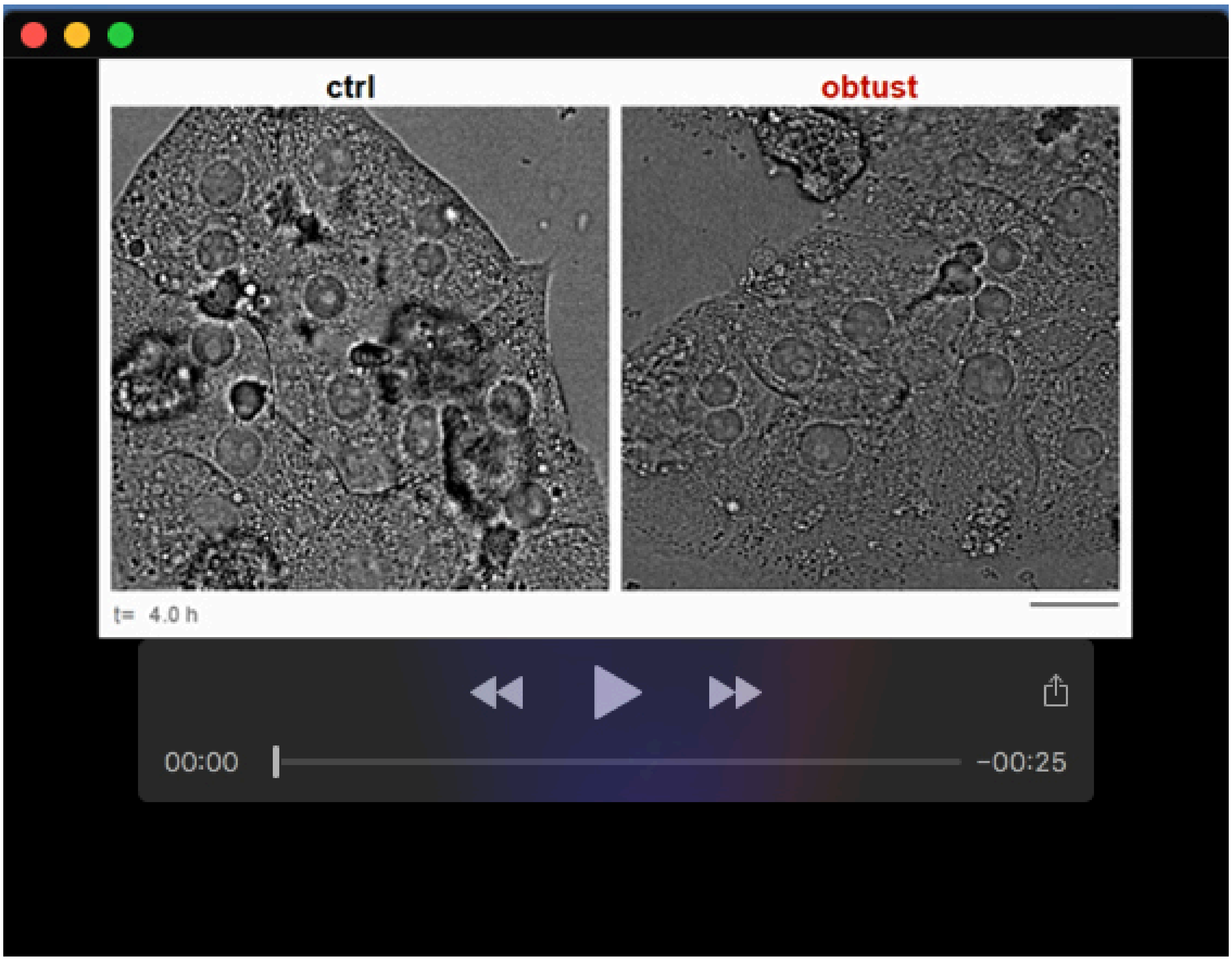

Movie 1. Obtustatin inhibits elongation of nascent BC
Time-lapse (5 fps) of control and obtust-treated hepatocytes plated on coll-coated glass during bile canaliculi formation induced by collagen overlay. Bar 20 µm.
